# Mentorship, Excellence, and Growth: A Reflection to Dr. Gold's Clinic

**DOI:** 10.1111/jocd.70513

**Published:** 2025-10-17

**Authors:** Mohamad Goldust

**Affiliations:** ^1^ Department of Dermatology Yale University School of Medicine New Haven Connecticut USA

**Keywords:** Dr. Gold's clinic, excellence, growth, mentorship

Dear Editor,

I am writing to express my deep gratitude for the incredible opportunity to complete a rotation at Dr. Gold's esteemed clinic in Nashville. My experience there was truly extraordinary and stands out as one of the most enriching moments of my dermatology career.

From the very first day, I was warmly welcomed by Dr. Gold and his dedicated team. The atmosphere in the clinic was not only highly professional but also incredibly supportive, collaborative, and inspiring. Dr. Gold's passion for both medical and aesthetic dermatology was evident in every patient interaction, teaching moment, and clinical discussion. His commitment to excellence and innovation left a lasting impression on me.

During the rotation, I had the privilege of observing and participating in a wide range of dermatologic procedures, from cutting‐edge aesthetic treatments to complex medical cases. The clinic's integration of the latest technologies and evidence‐based techniques provided me with invaluable insights into current advancements in both medical and aesthetic dermatology. Dr. Gold's willingness to share his extensive knowledge and clinical pearls, along with his honest perspectives on treatment outcomes, enhanced my learning experience.

However, my rotation was not just about clinical exposure. One of the most impactful aspects was gaining insight into the management of a high‐volume, multidisciplinary dermatology practice. I witnessed firsthand how efficiency, teamwork, and clear leadership contribute to the operation of a busy clinic. Dr. Gold's ability to lead with vision and compassion was truly inspiring. His emphasis on continued learning, patient satisfaction, and team cohesion offered me valuable lessons as both a physician and a future leader in our field.

Dr. Gold's mentorship was particularly meaningful to me. Despite his demanding schedule, he consistently took the time to explain concepts, encourage me, and challenge me to think critically. His feedback was constructive and thoughtful, and I left every interaction feeling motivated to continue growing as a clinician and as a person. It was clear that he is deeply invested in developing the next generation of dermatologists.

Meeting Dr. Gold and observing his work in his prestigious clinic was an absolute honor. He exemplifies excellence in dermatology and sets a benchmark for aspiring leaders in our specialty. The culture of innovation and excellence that he provides is something I hope to use in my future practice (Figure 1).

**FIGURE 1:**
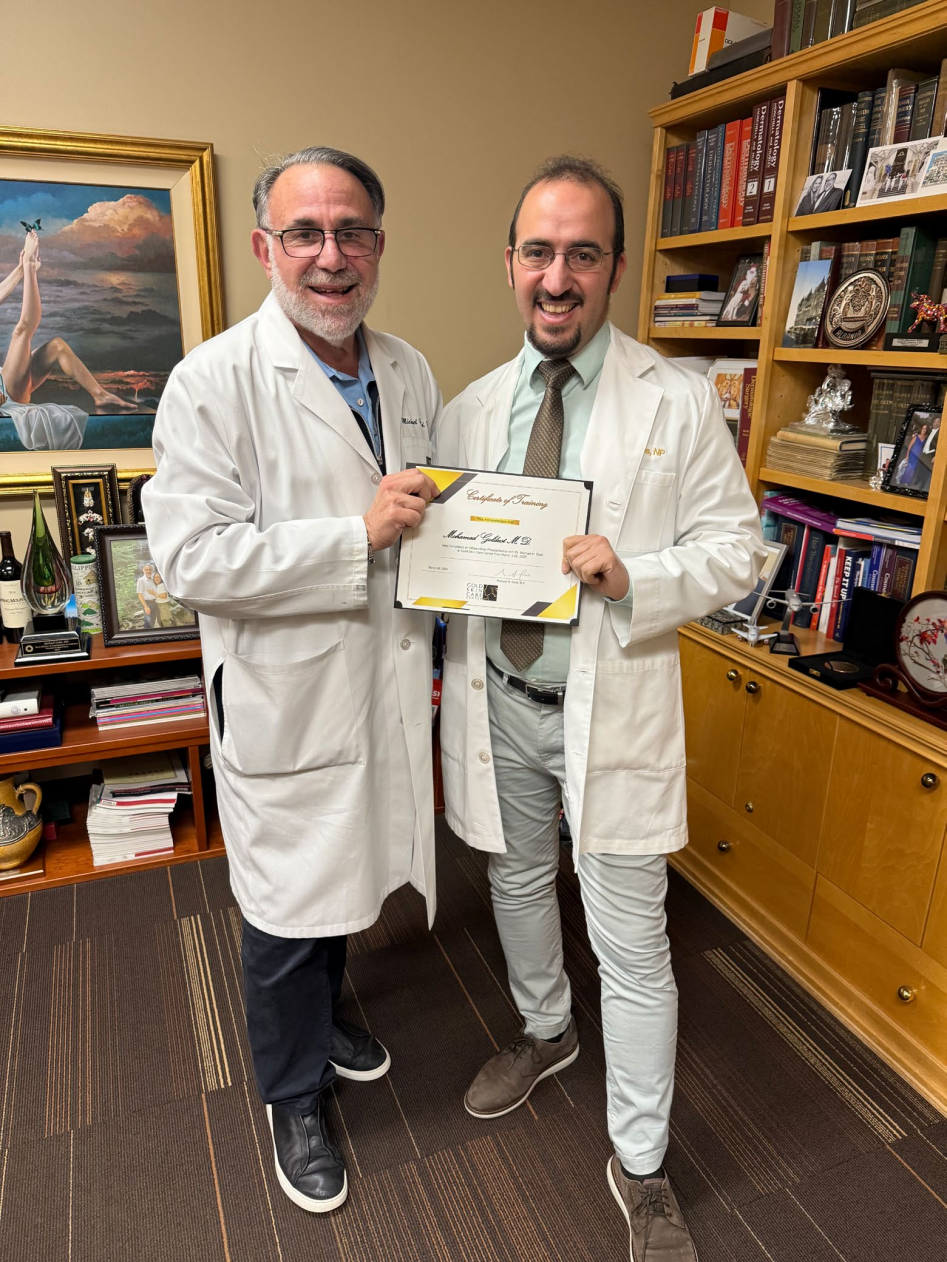
Honored to receive this Certificate of Training in recognition of my clinical and academic collaboration. Grateful for the mentorship, inspiration, and shared commitment to advancing dermatologic excellence.

In summary, my rotation at Dr. Gold's clinic was an incredibly rewarding experience both academically and personally. I left Nashville with a renewed sense of purpose, a deeper understanding of comprehensive dermatologic care, and a broader perspective on leading effectively in a fast‐paced clinical environment. I am truly grateful for this opportunity and look forward to applying what I have learned to my future endeavors in dermatology.

Thank you once again, Dr. Gold, for your generosity, mentorship, and for opening the doors of your remarkable clinic to me. This experience will undoubtedly remain a highlight of my professional journey.

## Disclosure

*Disclaimer*: “We confirm that the manuscript has been read and approved by all the authors, that the requirements for authorship as stated earlier in this document have been met and that each author believes that the manuscript represents honest work.”

## Consent

The author has nothing to report.

## Conflicts of Interest

The author declares no conflicts of interest.

## Data Availability

The author has nothing to report.

